# Differentiation of Urothelial Carcinoma into Two Distinct Subtypes, Glandular and Squamous, in Three Different Organs

**DOI:** 10.7759/cureus.7280

**Published:** 2020-03-15

**Authors:** Jose M El-Asmar, Moustafa Moussally, Nassib Abou Heidar, Wassim Wazzan, Jad A Degheili

**Affiliations:** 1 Division of Urology, Department of Surgery, American University of Beirut Medical Center, Beirut, LBN

**Keywords:** urinary bladder, renal pelvis, glandular and epithelial neoplasms, squamous cell neoplasm, transitional cell carcinoma

## Abstract

Urothelial carcinoma (UC) is a unique entity with different histological variants: squamous, glandular, small cell, micropapillary, sarcomatoid, and plasmacytoid. Each of those subtypes behaves differently. As such, and in many scenarios, an accurate histological diagnosis is of paramount importance to dictate the therapeutic approach. We hereby present a unique case of urothelial carcinoma that differentiated into two distinct histological subtypes, squamous and glandular, in three different organs within the genitourinary tract. We also describe the pathological and clinical differences entailed between the two histological variants in bladder and upper urinary tract urothelial carcinoma.

## Introduction

Urothelial carcinomas (UC) are known to exhibit different types of histological variations [1]. Each histologic variant behaves differently. An accurate histological diagnosis is crucial for the optimal management of the disease. Each variant is associated with a different therapeutic approach, and clinical outcomes differ accordingly [2]. We hereby report a unique case of urothelial carcinoma that differentiated into two distinct histological subtypes, glandular and squamous, in three different organs within the genitourinary tract. The uniqueness of this case lies in the presence of synchronous urothelial carcinomas in the genitourinary tract with distinct histological differentiation: glandular and squamous.

## Case presentation

This is a case of a 62-year-old gentleman with a medical history of hypertension and dyslipidemia who presented several years ago for multiple episodes of gross hematuria. Initial workup included an ultrasound, followed by an enhanced computed tomography scan with delayed phases (CT urography) revealing a 2 cm polypoid lesion arising from the left lateral wall of the bladder. Both kidneys and upper urinary tracts appeared free from filling defects with the absence of hydronephrosis. Following that, transurethral resection of the bladder wall tumor (TURBT) was done with a subsequent pathology of high-grade papillary urothelial carcinoma invading the lamina propria, with no involvement of the muscularis propria, consistent with a tumor, node, metastasis (TNM) staging of pT1 high grade. One month later, a second-look TURBT was negative for any residual carcinoma. As such, the patient was started on intravesical Bacillus Calmette-Guérin (BCG) therapy. A six-week induction course was followed by three-year maintenance.

**Figure 1:**
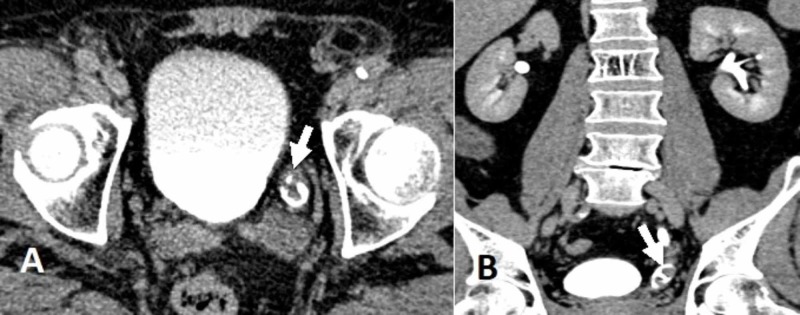
Enhanced computed tomography scan during the excretory phase (CT urography) (A) filling defect noted within the distal left ureter (arrow) on the axial view; (B) Coronal view showing a filling defect within the distal left ureter (arrow), suggestive of urothelial carcinoma

The patient was regularly followed up for three consecutive years with a yearly CT urography and diagnostic cystoscopies initially at three-month intervals and then at six-month intervals. There was no evidence of tumor recurrences, up until a follow-up CT urography scan three years after diagnosis demonstrated a small filling defect within the distal portion of the left ureter (Figure 1).

Retrograde pyelography with ureteroscopy was done; a polypoid lesion within the distal ureter was noted, measuring around 1.5 cm. A lesion biopsy revealed the presence of a low-grade noninvasive urothelial carcinoma. At the time, random bladder wall biopsies were negative for recurrence.

Taking into consideration his overall health status with a baseline serum creatinine level of 1.3 mg/dL, a multidisciplinary meeting decided to proceed with a left distal segmental ureterectomy with ureteral re-implantation into a Boari flap at the anterior aspect of the bladder wall. Pathological studies indicated the presence of a high-grade, noninvasive urothelial carcinoma of the ureter.

Throughout the next three years, the patient underwent four TURBTs, consistently revealing low-grade, non-muscle, invasive urothelial carcinoma, whilst bilateral upper urinary tract evaluations by ureteroscopy and retrograde pyelography were consistently negative.

**Figure 2:**
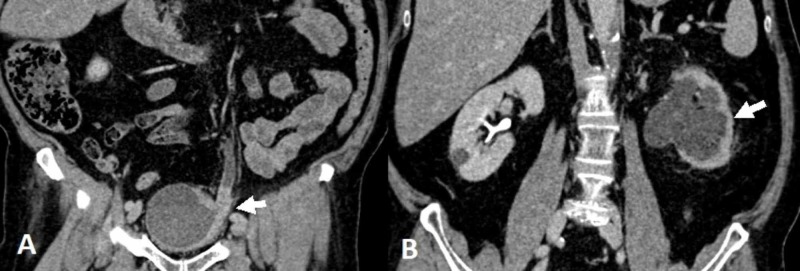
Enhanced computed tomography scan (A) Coronal view showing urothelial thickening at the level of ureteral re-implantation site (arrow), also noted is a left lateral bladder wall thickening; (B) Another coronal view of the upper tracts revealing a hydronephrotic left kidney, with effaced parenchyma and decreased uptake of contrast (arrow).

A year later, he presented with lethargy, nausea, and decreased PO intake. Acute onset of kidney injury with a serum creatinine level of 2.4 mg/dL, up from a previous level of 1.4 mg/dL, was noted. After adequate hydration and a drop of serum creatinine back to baseline, a follow-up CT urography scan was done and showed urothelial thickening along the left distal ureter, almost obliterating the ureteral lumen at the site of the previous re-implantation. Severe hydronephrosis and hydroureter were noted along with effaced kidney parenchyma (Figure 2).

Consequently, a cystoscopic evaluation with retrograde pyelography followed by ureteroscopy showed multiple sessile tumors at the left periureteral bladder wall with multiple fronds projecting out of the ureteral opening where the previous re-implantation was done. Complete resection of the bladder tumor was done, and a JJ stent was successfully inserted post resection of the fronds at the site of re-implantation. Pathological studies of the bladder wall tumor, as well as the ureteral fronds, revealed an invasive high-grade urothelial carcinoma (pT2). Postoperatively, his serum creatinine settled at 1.6 mg/dL.

**Figure 3:**
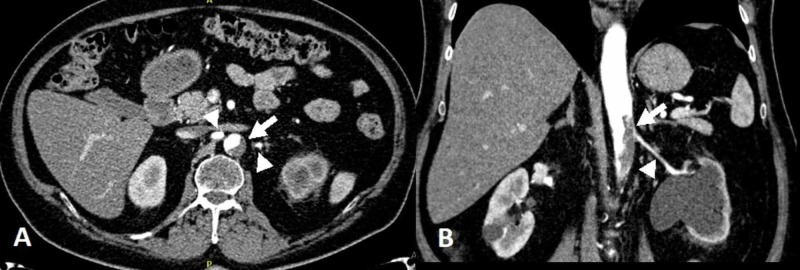
Enhanced computed tomography scan during the arterial phase (A) Axial view at the level of the takeoff of the renal arteries, revealing an aortic mural thrombus (arrow) almost obliterating the ostium of the left renal artery. Both renal arteries seemed opacified (arrowheads); (B) Coronal view showing again the mural aortic thrombus (arrow) at the level of the renal artery and extending caudad. Also noted here is the left renal artery (arrowhead), left hydronephrosis, and decreased uptake of the contrast due to effaced kidney parenchyma, unlike the contralateral right kidney.

Another multidisciplinary meeting decided to treat with an adjusted dosage of a platinum-based regimen of cisplatin and gemcitabine. His course was complicated by an abdominal aortic thrombus with 50% luminal narrowing at the level of the renal arteries (Figure 3).

As such, chemotherapy was halted and he was started on therapeutic anticoagulation. The panel’s decision was to proceed for aggressive surgical management involving a left radical nephroureterectomy, cystoprostatectomy, and an ileal conduit for urinary diversion.

**Figure 4:**
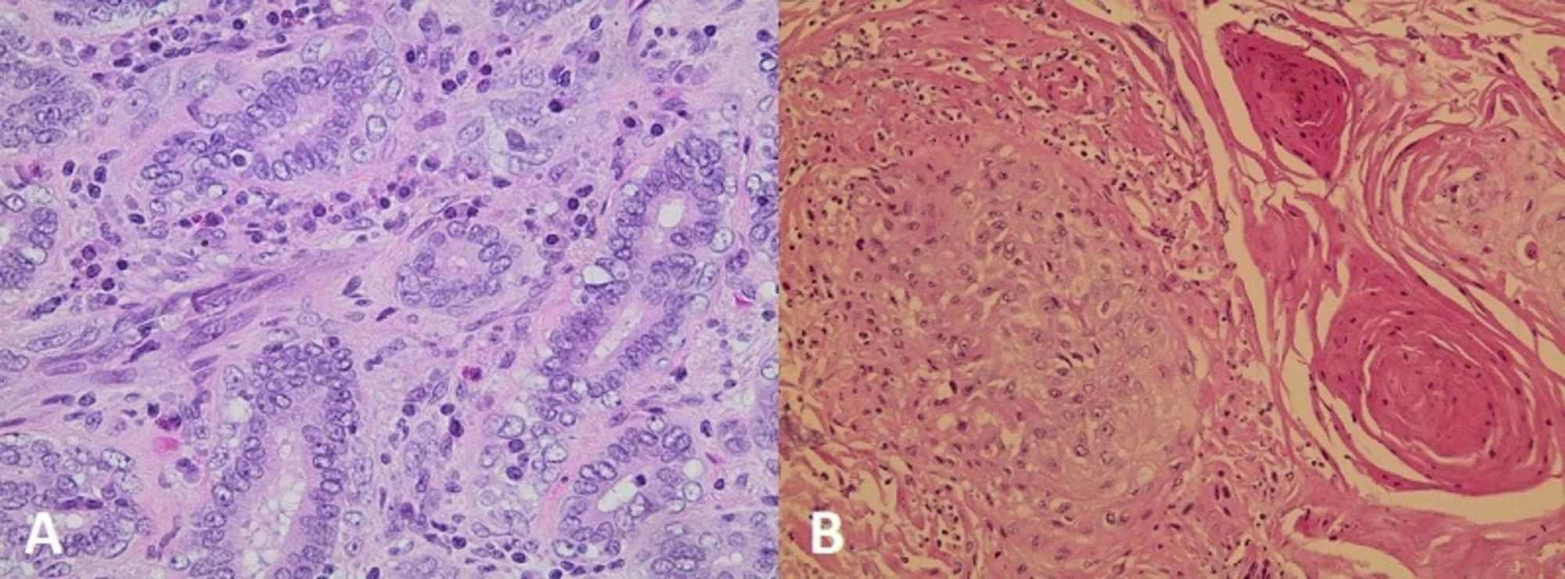
(A) Showing the photomicrograph of urothelial carcinoma of the distal left ureter with focal formation of glands (H&E, x400); (B) Showing squamous differentiation in urothelial carcinoma of the renal pelvis (H&E, x200)

Final bladder pathology at the anterior wall of the bladder revealed an infiltrating, high-grade urothelial carcinoma with glandular and squamous differentiation, whereas the distal portion of the left ureter revealed an infiltrating high-grade urothelial carcinoma with glandular differentiation. The left kidney pathology revealed an infiltrating high-grade urothelial carcinoma of the renal pelvis with squamous differentiation (Figure 4).

To note, the remaining part of the bladder wall showed superficial foci of high-grade urothelial carcinoma infiltrating the dense fibroconnective tissue with carcinoma-in-situ.

The patient’s postoperative course was complicated by acute kidney injury with serum creatinine reaching 9 mg/dL that was treated conservatively with adequate intravenous hydration. He was discharged on postop day 15 when his creatinine dropped back to his preoperative baseline of 1.6 mg/dL.

## Discussion

Urothelial carcinoma comprises around 90% of bladder tumors [3]. They exhibit a wide array of histological variants: squamous, glandular, small cell, micropapillary, sarcomatoid, and plasmacytoid [1]. In the case presented above, urothelial carcinoma with squamous differentiation was seen at the renal pelvis whereas urothelial carcinoma with glandular differentiation was seen at the distal ureter. Moreover, both subtypes were detected in the bladder. Identifying the correct histological variant is vital due to the various therapeutic approaches and clinical outcomes associated with each variant [4]. Furthermore, such variants possess a prognostic significance of the metastatic nature of the disease [4].

Urothelial carcinoma with squamous differentiation comprises approximately 60% of UC variants, whereas UC with glandular differentiation comprises approximately 10% of all UC [4]. Urothelial carcinoma with squamous differentiation is characterized by the presence of intercellular bridges or keratinization [1]. Two entities of squamous differentiation exist: partial or complete. Partial squamous differentiation, also referred to as UC with squamous differentiation, usually presents at a later stage. On the contrary, complete differentiation involves the entire tumor and is referred to as pure squamous cell carcinoma of the bladder. It is usually associated with chronic catheterization and schistosomiasis infection. On histology, differentiating between UC with squamous differentiation and pure squamous cell carcinoma of the bladder is a challenging yet necessary diagnosis, as the therapeutic approach is tailored accordingly [4-5].

Urothelial carcinoma with glandular differentiation is characterized by the presence of enteric gland-like or intratumoral tubular spaces [6]. Glandular differentiation exists either as pure adenocarcinoma of the bladder or UC with glandular differentiation. Whilst pure adenocarcinoma of the bladder is a rare entity, it is also thought to be associated with schistosomiasis as well as bladder exstrophy [5]. Similarly, determining the exact tumor histology is essential in determining the best treatment modality [4-5].

The uniqueness of this case lies in the presence of synchronous urothelial carcinomas in the genitourinary tract with distinct histological differentiation: glandular and squamous. The presence of different histological variants within the same patient is associated with a worse prognosis [1-2]. Moreover, the presence of urothelial carcinoma with divergent differentiation, specifically a glandular and/or a squamous one, has been linked to higher tumor stages at presentation [2,6-9]. In addition, it has been linked with a more aggressive disease course [2,9-10]. In fact, their presence has been found to be an independent predictor of high recurrence rates and poorer cancer-specific survival (CSS) in both urothelial carcinoma of the bladder and upper tract urothelial carcinoma, respectively [8-11]. Nevertheless, some studies revealed that the prognosis of these rare entities is yet to be defined. A systematic review and meta-analysis conducted by Chen et al. concluded that the variant histological patterns of urothelial carcinoma of the bladder do not portend poorer prognosis [12].

Patient outcomes in pure urothelial carcinoma of the bladder are not significantly different from those of patients with glandular and/or squamous differentiation [6]. In addition, a study conducted by Tang et al. revealed no significant difference, in terms of CSS, between patients with a single variant of upper urinary tract urothelial carcinoma and an upper urinary tract urothelial carcinoma with both squamous and glandular differentiation. Whereas, patients with squamous and/or glandular differentiation had significantly worse cancer-specific survival than pure upper tract urothelial carcinoma patients [9].

Early radical cystectomy remains the definitive treatment of choice for patients with a T2 tumor stage or higher disease of pure or variant UC [5]. However, recent studies highlighted the role of trimodal bladder sparing protocol with maximal TURBT, chemotherapy, and radiation therapy in the treatment of variants of UC [3,5]. Moreover, histological variants play a major role in tailoring therapeutic approaches to cT1 disease and predicting response to neoadjuvant chemotherapy [5].

## Conclusions

In conclusion, urothelial carcinomas are tumors capable of divergent differentiation. The identification of the various histologic variants is essential in determining therapeutic approaches.
